# CardioHotspots: a database of mutational hotspots for cardiac disorders

**DOI:** 10.1093/database/baae034

**Published:** 2024-05-15

**Authors:** Alberto García S, Mireia Costa, Alba García-Zarzoso, Oscar Pastor

**Affiliations:** PROS Research Center, VRAIN, Polytechnic University of Valencia, Camino de Vera S/N, Valencia 46022, Spain; PROS Research Center, VRAIN, Polytechnic University of Valencia, Camino de Vera S/N, Valencia 46022, Spain; PROS Research Center, VRAIN, Polytechnic University of Valencia, Camino de Vera S/N, Valencia 46022, Spain

## Abstract

**Mutational hotspots are DNA regions with an abnormally high frequency of genetic variants. Identifying whether a variant is located in a mutational hotspot is critical for determining the variant’s role in disorder predisposition, development, and treatment response. Despite their significance, current databases on mutational hotspots are limited to the oncology domain. However, identifying mutational hotspots is critical for any disorder in which genetics plays a role. This is true for the world’s leading cause of death: cardiac disorders. In this work, we present CardioHotspots, a literature-based database of manually curated hotspots for cardiac diseases. This is the only database we know of that provides high-quality and easily accessible information about hotspots associated with cardiac disorders. CardioHotspots is publicly accessible via a web-based platform (** https://genomics-hub.pros.dsic.upv.es:3099/).

**Database URL**: https://genomics-hub.pros.dsic.upv.es:3099/.

## Introduction

Precision medicine is a medical paradigm that aims to provide patients with personalized diagnoses and treatments based on their specific characteristics (1). One of the cornerstones of precision medicine is genetics, which studies the effect of variants in a patient’s DNA sequence on disorder susceptibility or treatment response capabilities.

The process of determining the effect of a DNA variant on a patient’s health status is called variant interpretation. Variant interpretation is a complex process in which various factors, such as the variant’s population frequency, its impact on the protein sequence or specific characteristics of the DNA region affected, are evaluated and weighted (2).

One of the several factors considered when interpreting variants is whether they are located in a mutational hotspot. Mutational hotspots are DNA regions where genomic variants are detected at an abnormally high frequency (3). These regions tend to have a higher concentration of disease-causing variants and, as a result, are a moderate predictor of pathogenicity for those variants located in them (2).

Hotspot research is nowadays heavily focused on oncology, as variants found in hotspots are well known to contribute to tumor development and growth (4). Thus, databases such as Cancer Hotspots (https://www.cancerhotspots.org) (5, 6) have emerged in recent years to provide knowledge about these DNA regions in the context of oncology disorders.

However, knowledge about hotspots is crucial for any disorder where genetics plays an important role. This is the case with cardiac disorders, which are the leading cause of death worldwide (7). They frequently have a genetic origin and thus are inherited. Studying the genetic basis of these disorders is becoming increasingly valuable for diagnosing them early, preventing sudden cardiac death and selecting appropriate treatments. However, no centralized database of high-quality clinical knowledge about hotspots associated with cardiac disorders is available to clinical experts. Instead, this information is scattered across hundreds of scientific articles.

This work presents CardioHotspots, a database of hotspots for cardiac disorders that aims to cover the needs of the above-mentioned clinical experts. Since relevant information is scattered across hundreds of scientific articles, the body of knowledge of CardioHotspots was obtained through an expert literature review. CardioHotspots is publicly available through a web-based platform that offers the following functionalities:

- Visualize hotspots along with protein sequence features: Each protein affected by a mutational hotspot has its entire sequence displayed. Sequence features, such as domains, binding sites and hotspots, are depicted along this sequence for easy and intuitive visualization.
- Visualize hot spot information: The information associated with each hot spot is displayed on a table with sorting and filtering capabilities.
- Download the data: The full list of hotspots associated with cardiac disorders and their associated information has been made available for download in comma-separated values (CSV) format.

## Materials and methods

### Identification of relevant information

The information regarding hotspots in cardiac disorders is characterized by its dispersion across hundreds of scientific articles and the consequent heterogeneity because of this dispersion. Conceptual modeling has proven to be an effective and efficient solution to deal with this heterogeneity. Moreover, conceptual modeling techniques allow for improved communication and knowledge sharing, more efficient integration of dispersed information and better harmonization of heterogeneous information (8–10).

**Figure 1.**
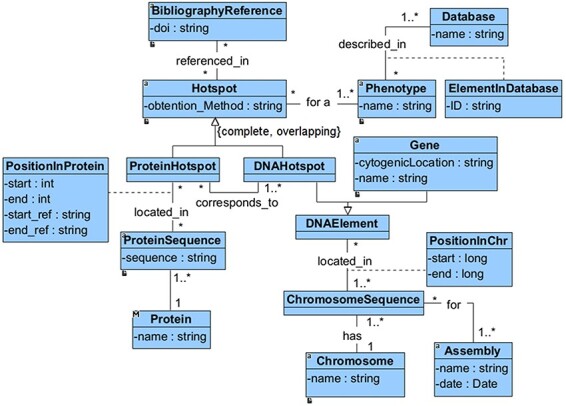
Conceptual model of CardioHotspots.

We have defined a conceptual model to drive the extraction, integration and harmonization of the heterogeneous information associated with hotspots. This model, depicted in Figure 1, comprises all of the relevant information that is required to characterize a hotspot. It also includes information about who, how and when the hot spot was identified.

The main concept of this model is the ‘Hotspot’, which can be a ‘DNAHotspot’ if it is described at the DNA level or a ‘ProteinHotspot’ if it has been described at the protein level. This specialization is represented as complete and overlapping because the identified hotspots exist at both the DNA and protein levels. Each ‘Hotspot’ is obtained using a method (‘obtention_method’) used to identify the hotspot, such as literature research and DNA sequencing assay. Besides, the hotspot must be associated with at least a ‘BibliographyReference’.

Both the ‘DNAHotspot’ and the ‘ProteinHotspot’ location (‘PositionInProtein’ or ‘PositionSequence’) depend entirely on which gene or protein sequence is being considered. A ‘DNAHotspot’ is located in one or many chromosomal sequences (every ‘ChromosomeSequence’ must refer to a specific genomic ‘Assembly’), while a ‘ProteinHotspot’ is located in one or many protein sequences. According to our representation, a DNAHotspot overlaps a gene if it is located within its range. Finally, every ‘Hotspot’ is described in the context of at least one ‘Phenotype’, which must be described in a specialized ‘Database’.

This conceptual model enabled us to comprehend the domain and correctly and efficiently integrate all of the information related to mutational hotspots. The processing and integration of the data (as reported in the following sections) is a complex process that relies on this conceptual model. The richness of this model opens the tool up to future extensions and enhancements, such as the ability to perform complex queries. Despite this, the data available for download conceal this fragmentation and provide the user with a simple and consolidated representation of the data.

### Data collection and curation

**Figure 2.**
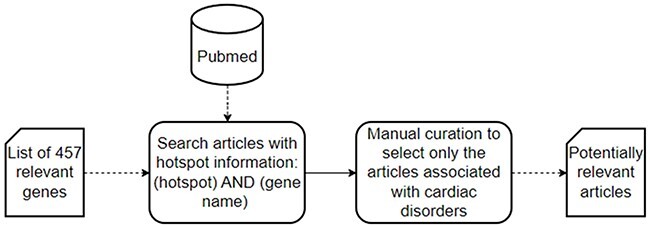
A schematic representation of the data collection process used to collect the set of potentially relevant articles.

The data collection process consists of two steps (Figure 2): first, a literature search of articles with hotspot information in PubMed, a specialized repository of biomedical literature (https://pubmed.ncbi.nlm.nih.gov); and second, a manual curation to select only those articles explicitly associated with cardiac disorders.

In the first step, we searched PubMed for articles that could contain information about hotspots in the context of cardiac disorders. We searched literature describing hotspots affecting genes or proteins associated with cardiac disorders. Together with our clinical partners (Hospital La Fe and Hospital General de Alicante, in the Comunitat Valenciana, Spain), we identified 457 relevant genes related to cardiac disorders, which were used in the following PubMed search: ‘(hotspot) AND (gene name)’.

Since a gene can be associated with multiple disorders, there may be articles not necessarily associated with cardiac disorders in the list of relevant articles obtained from the first step. Thus, the second step was a data curation process in which articles whose scope was not cardiac disorders were discarded via manual curation of abstracts. As a result, we obtained an updated list of potentially relevant articles that solely focus on cardiac disorders.

After the data collection process finished, we curated each article to get all the information deemed relevant for characterizing a hotspot according to the conceptual model defined above. We used complementary tools to complete the missing data if the required information was unavailable. Table 1 describes the complementary tools used and its concrete purpose. If the missing information could not be obtained with these tools, the article was discarded and thus not included in the CardioHotspots knowledge base.

**Table 1.** Description of the complementary tools used in CardioHotspots

Tool	Description	Purpose
RefSeq	Database of genomic, transcript and protein sequences (11).	Determine the position of the exon in chromosome genomic sequences.
SynVar	Tool for generating synonym Human Genome Variation Society expression for representing variant positions in different reference sequences (12).	Obtain the variant position with respect to the canonical reference sequence when it is not available in the article.
UniProt	Database containing high-quality information about proteins (13).	Determine the position of the hotspot regarding the canonical protein sequence.
ClinVar	Database containing information about variants and their relation with human health (14).	Obtain basic information such as the gene affected, protein position and the position in different assemblies.

Finally, we standardized the collected information associated with the articles by mapping attribute values expressed as free text into the corresponding ontology terms. This mapping is particularly relevant for achieving semantic interoperability. Semantic interoperability is a cornerstone for maximizing the added value of information and data artifacts, and it cannot be achieved without the support of ontologies (8). First, we used the Mondo Disease Ontology (MONDO) (15) and human phenotype (16) ontologies to standardize phenotype information associated with the hotspot (i.e. the ‘name’ attribute of the ‘Phenotype’ class). Then, the method used to identify the hotspot (‘obtention_method’ attribute of the ‘Hotspot’ class) was standardized using the evidence & conclusion (17) and biomedical investigation (18) ontologies. After the mapping process, the data were included in the CardioHotspots database.

### Development tools and implementation

CardioHotspots is a novel, user-friendly database that provides up-to-date information about hotspots associated with cardiac disorders. JavaScript and the React framework were used to generate the CardioHotspots website. The Ideogram (v1.41.0) library is used for chromosome visualization, and the seqparse (v0.2.0) and seqviz (v3.7.6) libraries are used for depicting the circular and linear sequence viewers. In addition, the ag-grid package (v29.0.0) is used to implement the table containing the hotpots and their associated information. The webpage is hosted in a node.js server using Express.js (v4.18.2).

## Results

### Database statistics

The data collection and curation process started with a list of 457 genes identified by cardiology experts. After searching PubMed for articles describing hotspots in those genes, we only identified articles for 145 of the 457 original genes, as shown in Figure 4. These articles were filtered to include only those about cardiac disorders. As a result, only 60 genes remained with potentially relevant articles (Figure 4).

**Figure 3.**
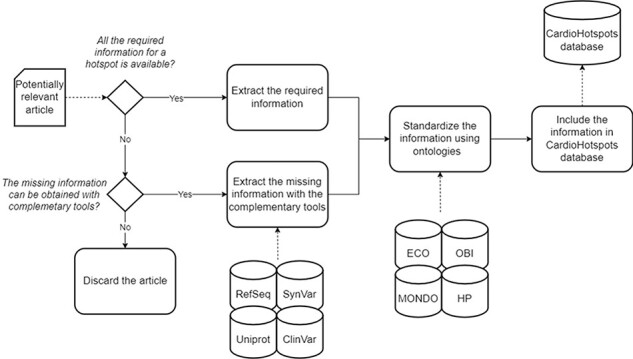
A schematic representation of the data curation process used to generate the CardioHotspots database.

These 60 genes accumulated a total of 106 articles, which were curated following the workflow described in Figure 3 (see Figure 5 for a detailed result of the curation process). Only 49 of the original 106 articles (i.e. 46.22%) were ultimately considered for inclusion in the CardioHotspots database. The most common reason for discarding an article was that it lacked all of the necessary information to characterize a hotspot (33 articles, 31.1% of the total), and we could not complete the missing information with the complementary tools described earlier. Besides, 10 articles were discarded because they described recombinational hotspots, a different type of hotspot that is out of our database’s scope. We discarded 14 more articles because they were not ‘free to access’, and the information available could not be processed.

**Figure 4.**
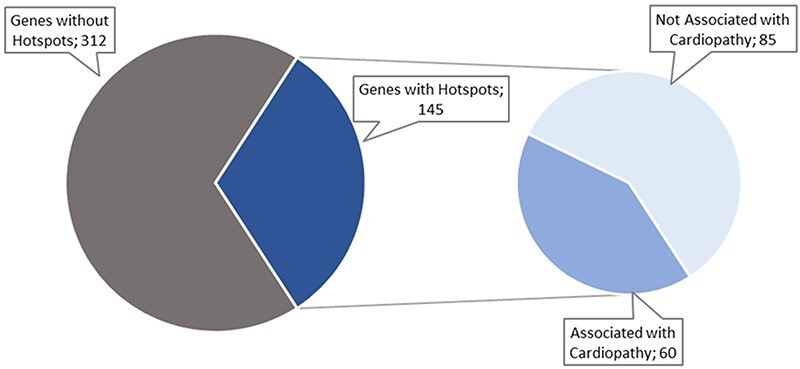
Hotspots distribution per gene.

**Figure 5.**
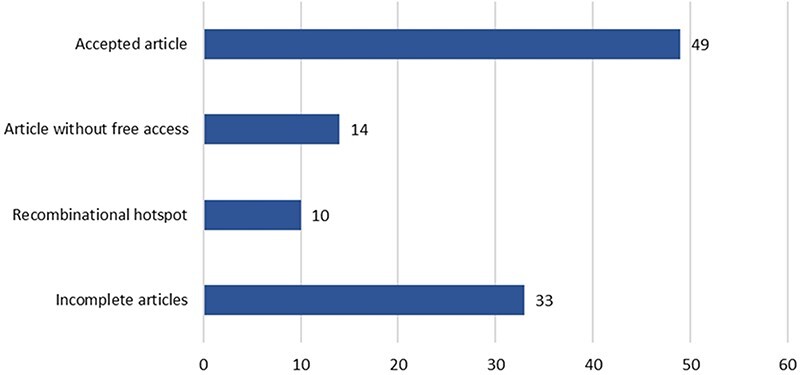
Article processing results.

**Figure 6.**
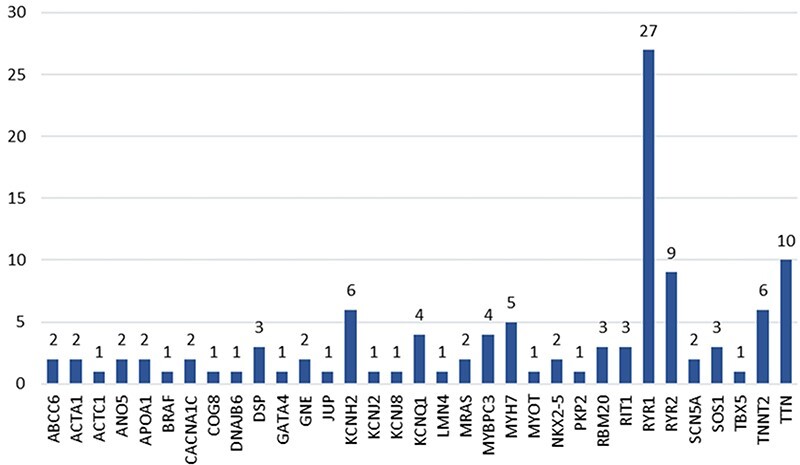
Distribution articles per gene.

The 49 accepted articles described a total of 113 hotspots located in 33 genes (Figure 6). The gene with the highest number of hotspots (i.e. 27 hotspots) is RYR1. This gene codes for the ryanodine receptor protein in the striated muscle, which acts as a calcium release channel (https://www.ncbi.nlm.nih.gov/gene/6261). The TTN gene, which codifies for a protein with vital functions in the structure of the heart muscle (https://www.ncbi.nlm.nih.gov/gene/7273), is the second gene with the highest number of hotspots (i.e. 10 hotspots). The remaining genes have less than 10 hotspots associated and most commonly having between 1 and 2 hotspots associated.

Each of the 113 hotspots is associated with one or more cardiac disorders. With 24 hotspots associated, the ‘Malignant hyperthermia, susceptibility to, 1’ (MONDO:0007783) is the most common disorder. This disorder affects the skeletal muscle and can produce tachycardia, cardiac arrhythmia or even cardiac arrest (19). The second most common disorder is ‘Hypertrophic Cardiomyopathy’ (MONDO:0005045), a heart condition characterized by increased ventricular wall thickness (20). Another disorder with a high number of hotspots (i.e. 16) is ‘Catecholaminergic polymorphic ventricular tachycardia’ (MONDO:0017990), which affects the electrical activity of the heart, causing syncopes with exercise and fast ventricular tachycardias (21). All of the other cardiac disorders have less than nine hotspots associated.

### User interface

CardioHotspots web application is available at the following URL: https://genomics-hub.pros.dsic.upv.es:3099/. The application comprises four sections: (i) ‘main page’, (ii) ‘hotspots’, (iii) ‘about’ and iv) ‘download’.

**Figure 7.**
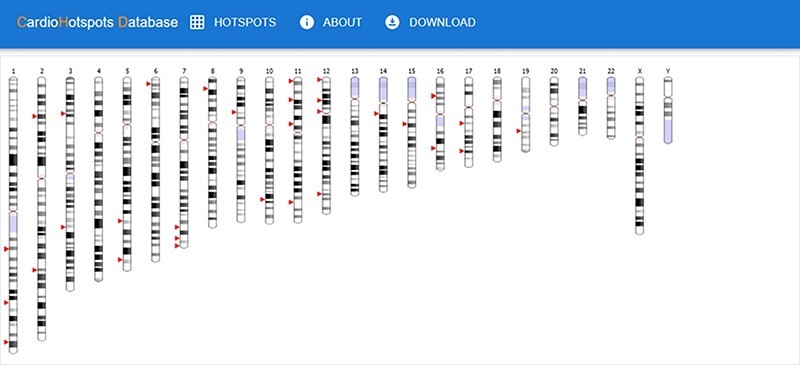
CardioHotSpots user interface (UI) showing genes with hotspots.

**Figure 8.**
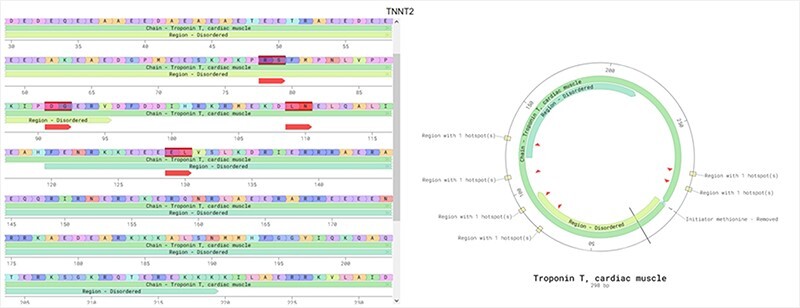
CardioHotspots ProteinViewer.

The ‘main page’ is depicted in Figure 7. Initially, this page shows an idiogram highlighting the genes with at least one hotspot by depicting a red arrow. This representation aims to provide a visual overview of specific genomic regions where mutational hotspots associated with cardiac disorders exist, thus helping identify potential areas of interest. When the user hovers an arrow, the name of the gene and its chromosomal position are displayed. Clicking on one of these arrows visually represents all the hotspots affecting the selected gene using two sequence viewers. More specifically, this new visualization depicts the canonical sequence of the protein codified by the selected gene in both linear and circular form, highlighting any critical region of the protein in different colors (e.g. domains or binding sites) and the hotspots in that gene, depicted in red. Figure 8 shows an example of this representation for the TTN gene.

**Figure 9.**
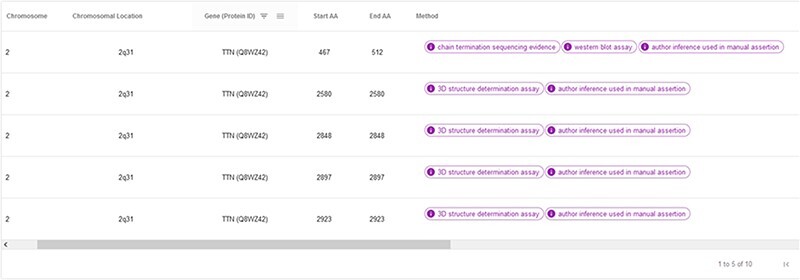
Example of the table displayed on the ‘Hotspots’ page.

The ‘hotspots’ page consists of a table summarizing the most relevant information of the CardioHotspots database (Figure 9). The columns selected for this table represent specific attributes of the CardioHotspots conceptual model (Figure 1). Table 2 contains a description of each column as well as the attribute of the conceptual model to which it refers.

**Table 2.** The list of columns in the Hotspots page table

Name of the column	Description	Conceptual model representation
Chromosome	Chromosome where the hotspot is located.	Chromosome class—name attribute
Chromosomal location	Cytogenic location of the gene affected by the hotspot.	Gene class—cytogenicLocation attribute
Gene (Protein ID)	Gene name and protein ID in the UniProt database.	Gene class—name attribute Protein class—ID attribute
Start AA	Start of the hotspot in the canonical protein sequence.	PositionInProtein class—start attribute
End AA	End of the hotspot in the canonical protein sequence.	PositionInProtein class—end attribute
Method	Method used to obtain the hotspot.	Hotspot class—obtentition_Method attribute
Phenotype	Name of the associated phenotype.	Phenotype class—name attribute
DOI	DOI of the publications where the hotspot was described.	BibliographyReference class—doi attribute
Comments	Additional information for the hotspot, such as if it has been described in the context of a particular population, is described here.	Not represented in the model

The ‘About’ page contains information about the experts who developed CardioHotspots database and the funding under which this project has been developed. Finally, the ‘Download’ page allows the user to download a CSV file that contains the same information represented in the table of the hotspots section.

## Conclusions and future outlook

### Utility of the database for researchers, clinicians and patients

The precision medicine concept emerged more than 20 years ago, and its first application was in the oncology domain (22). However, more and more specialties have adopted the personalized approach that precision medicine proposes over the years. One of these specialties is cardiology, where precision medicine has been proven effective for preventing, diagnosing and treating cardiac disorders. Therefore, genetic analysis is becoming a common clinical practice in this area, where even a new medical specialty called cardiogenetics has been proposed (23). CardioHotspots, with its representation of hotspots linked to cardiac disorders, is a significant step forward in the precision medicine application by all the geneticists and cardiologists applying genetic testing in this domain. Based on current knowledge, CardioHotspots has the potential to simplify data access and facilitate exploration, analysis and hypothesis generation about hotspots for cardiac disorders. Moreover, it allows for downloading the data in a format (CSV file) that allows for an easy integration with variant annotator software. Hereditary heart disorders affect a significant portion of the population, with an incidence of up to one in 200 (24). The prevalence is significantly higher when all heart problems are included, not only inherited ones. For this large number of potential patients, the database provides a resource with relevant information about cardiac disorders that can lead to better disease identification and treatment. Overall, the CardioHotspots database is a centralized hub for data about hotspots associated with cardiac disorders, providing a comprehensive and up-to-date single point of truth supporting clinical decision-making.

### Uniqueness and relevance of the CardioHotspots database

CardioHotspots is, to the best of our knowledge, the first database that provides information about hotspots in the domain of cardiac disorders. While there are other data sources about hotspots, their primary focus is oncology. Compared to other accessible hotspot data sources, CardioHotspots provides significant information regarding the hotspot’s genomic and protein location, the technique of acquisition and the specific set of disorders associated with that hotspot. In addition, CardioHotspots employs a conceptual model for data organization as well as ontologies for data standardization. This improves data integration, domain knowledge and interoperability of CardioHotspots data with other genomic data sources. Finally, end users can access information regarding hotspots and important protein backgrounds, such as whether the hotspot is located within a functional domain, via the CardioHotspots user interface. This, combined with quick access and download, enables users to exploit the information easily.

### Future updates of the database

When a new article regarding hotspots in the cardiac diseases domain is published, CardioHotspots should be updated. However, manually performing this task is error-prone and time-consuming. To address this issue, we are developing an Artificial Intelligence-based curation process that will include the following steps:

- Identify potentially relevant articles about hotspots that have been published.
- Choose only those related to specific diseases of interest.
- Retrieve relevant information from the selected articles and supplement it with complementary tools.

We created a Python-based prototype that executes Steps 1 and 2. Step 1 automates the retrieval of articles indexed by PubMed using the search specified in the Data Collection and Curation section. Step 2 uses Pubtator, a tool developed by the National Center for Biotechnology Information that uses natural language processing, to automatically identify biomedical entities mentioned in a given publication (25). Using this tool, we identified all of the diseases mentioned in the articles retrieved in Step 1. With this identification, we can select publications referring to a specific domain, which, in this case, is cardiac disorders. We evaluated the functionality of this prototype by searching for publications associated with the field of cardiac disorders. Starting from the initial 49 publications that our manual curation considered relevant (see Results section), our prototype identified 19 of the 49 articles, with an accuracy of 38.77%. Considering that the prototype is still in its early stages of development, these findings are encouraging and suggest that it will be a convenient tool for supplementing easing and making the manual curation process more efficient. Efforts to increase accuracy will include using Pubtator to identify potential hotspot mutations mentioned in publications.

The prototype is generic and can be reused for other disease domains. This will enable us to apply our approach in other domains that may benefit from hotspot knowledge in the future. This prototype’s code is available in (25). This new method will ensure that CardioHotspots provides accurate information in an automated manner.

CardioHotspots provides information derived from original articles with no quality processing. However, some factors, such as the method of acquisition, the number of years since the article was published or the number of citations, could be used to assess the reliability of the information in the future. Future work will also focus on implementing this reliability metric, which can be extremely useful for CardioHotspots users.

### Limitations of CardioHotspots

We intend to address some limitations in future updates to CardioHotspots. The first limitation is that we only consider PubMed to retrieve literature about hotspots associated with cardiac disorders. While PubMed is the most widely used source of biomedical literature, other sites such as Google Scholar, Scopus or the Web of Science may contain relevant papers that would not be retrieved using this approach. Another limitation of using only PubMed is that we can only access publications published in English. Other websites offer valuable literature in other languages that may be useful for specific demographics (e.g. the Scientific Electronic Library Online reports information in Spanish). Future work will include additional literature sites to CardioGraph in addition to PubMed.

Besides, the results are only available in CSV format. We plan to provide files compatible with the most commonly used variant annotation tools, allowing for a more direct integration than the CSV file allows.
