# Correction to: CardioHotspots: a database of mutational hotspots for cardiac disorders

**DOI:** 10.1093/database/baaf014

**Published:** 2025-02-17

This is a correction to: Alberto García S, Mireia Costa, Alba García-Zarzoso, Oscar Pastor, CardioHotspots: a database of mutational hotspots for cardiac disorders, *Database*, Volume 2024, 2024, baae034, https://doi.org/10.1093/database/baae034

Acknowledgement of funding support from the Generalitat Valenciana through the CoMoDiD project (CIPROM/2021/023) was omitted from the version originally published, and has been added.

