# Correction to: CardioHotspots: a database of mutational hotspots for cardiac disorders

**DOI:** 10.1093/database/baaf050

**Published:** 2025-09-25

This is a correction to: Alberto García S, Mireia Costa, Alba García-Zarzoso, Oscar Pastor, CardioHotspots: a database of mutational hotspots for cardiac disorders, *Database*, Volume 2024, 2024, baae034, https://doi.org/10.1093/database/baae034.

The funding section has been updated to acknowledge support via the pre-doctoral grant ACIF/2021/117.

